# Increasing Vaccination Awareness for Italian Primary Care Pediatricians: Game Design and Usability Study

**DOI:** 10.2196/70049

**Published:** 2025-11-03

**Authors:** Federico Marchetti, Martino Barretta, Antonio Di Mauro, Marco Bona, Chiara Amerighi, Antonio D'Avino

**Affiliations:** 1 GSK, Viale dell'Agricoltura 7, Verona, 37135, Italy, 39 0457741111; 2 Federazione Italiana Medici Pediatri, Crotone, Italy; 3 Federazione Italiana Medici Pediatri, Bari, Italy; 4 Choralia Comunicazione Interna e Formazione Srl, Milan, Italy; 5 Federazione Italiana Medici Pediatri, Naples, Italy

**Keywords:** primary care pediatricians, health education, immunization, meningococcal serogroup B, vaccination

## Abstract

**Background:**

Invasive meningococcal disease has a high fatality rate and can lead to severe long-term health issues. In Europe, serogroup B meningococcal disease (MenB) accounts for over half of invasive meningococcal disease cases. In Italy, MenB vaccination is recommended for all newborns, but the uptake is below the Ministry of Health Vaccination Plan target (uptake: 80.91%; target: 90%).

**Objective:**

A vaccine-based digital educational tool, Meningioca, was designed to increase primary care pediatricians’ (PCPs’) knowledge on the value and proper timing of MenB vaccination and to support communication of this value to parents.

**Methods:**

Meningioca was developed using Articulate, an authoring software, and released via the TalentLMS online platform. Players engaged in a sequence of activities and mini-games, taking on the role of a PCP and progressing through 7 modules. Each module corresponds to a different age group and follows a fixed sequence of topics, simulating typical discussions that might occur during health checks for each specific age group. At the launch, members of the Italian Federation of PCPs were invited to play the game via an email link and rated the game based on aspects such as overall enjoyment, the difficulty of modules, and usefulness of the game as a teaching tool.

**Results:**

Between March 2023 and May 2024, 471 PCPs accessed Meningioca, completing 1206 modules and 482 hours of learning. Meningioca received a mean rating of 4.4/5 (5 being the highest score), with many participants noting that they would recommend Meningioca to a colleague. “Communicating with the parent” and “health checks” were voted as participants’ favorite module topics; “general culture” and “child growth and development” were voted as the most difficult. At the end of Module 3, 75% (n/N=50/67) of players agreed that Meningioca was a teaching tool to refresh and strengthen knowledge to a high or very high extent.

**Conclusions:**

User feedback from Meningioca’s first year suggests that the game is enjoyable and a potentially valuable learning tool for increasing PCPs’ knowledge on vaccination.

## Introduction

Invasive meningococcal disease (IMD) is caused by *Neisseria meningitidis*. Despite its low incidence in Europe (0.1 cases per 100,000 individuals), IMD is a public health concern due to its high fatality rate (13% in infants aged <1 y) and severe long-term health implications [1].

In Europe, serogroup B meningococcal disease (MenB) is the leading cause of IMD, responsible for more than 50% of serogroup-documented cases. Recent national surveillance data from Italy also highlight MenB as the most common cause of IMD in the country [1,2]. MenB vaccination in Italy is offered as a 2-dose primary course (starting from ≥2 mo of age and subsequent dose 2 mo later) and 1 booster dose (at 13‐14 mo of age; a 2+1 schedule) [3]. However, MenB vaccination is recommended, not mandatory, in Italy [4] and nonmandatory vaccinations may be viewed as less important than mandatory vaccinations by citizens and health workers [5].

Due to the high incidence of invasive MenB cases in individuals aged under 1 year, studies analyzing both Italian and UK cohorts have highlighted the importance of starting a vaccination schedule as early as possible [6-8]. In two retrospective, observational studies, MenB vaccination in children aged <6 years was shown to be highly effective at preventing the disease [7,8]. However, despite the proven efficacy of MenB vaccination, mean MenB vaccination coverage in Italy for the 2020 birth cohort was reported to be 80.91% and therefore, lower than the Italian Ministry of Health’s target of 90% [3,9].

In Italy, primary care pediatricians (PCPs) are expected to perform scheduled health checks, recommend vaccinations, and verify the vaccination status of children. In a survey conducted by the Italian Federation of Pediatricians (FIMP) in 2019, out of over 1000 responses, approximately 80% of PCPs said they stress the importance of vaccinations during health checks, but nearly half said they do not proactively arrange such health checks [10]. Although it is widely understood that vaccination is important for disease prevention, parental vaccine hesitancy is a known barrier to adherence to childhood vaccinations, with reasons for vaccine hesitancy including concerns about the busy pediatric vaccination schedule, vaccine effectiveness, and vaccine safety [11]. Between below-target vaccination coverage and vaccine hesitancy among parents, there is a need for effective communication about the value of recommended vaccinations in order to increase vaccine uptake [12].

Serious games and gamification allow participants to learn in an experiential, learning-by-doing way through making content more engaging, facilitating improvement of knowledge, and offering a measure of progress to create healthy competition, encouraging participants to learn more [13]. The use of serious games to improve knowledge and skills of health care professionals has been shown to be as effective as traditional learning methods, such as learning from written text or lectures [13]. In the context of vaccines, digital gamification has been suggested to hold potential to increase vaccination knowledge and thereby coverage. The use of gamification to enhance vaccine uptake is a rapidly expanding field [14,15]; a scoping review published in 2020 found only 7 peer-reviewed articles evaluating gamified digital tools to increase vaccine knowledge; a similar study published in 2024 identified 28 such articles, a four-fold increase [14,15].

The aim of this project was to develop a digital gamified educational training tool, Meningioca, to inform PCPs of the value and proper timing of vaccination with a focus on MenB and to improve communication of this value to parents and caregivers.

## Methods

### Game Concept

Meningioca was designed in partnership with the FIMP, which comprises of over 5300 Italian PCPs (the target audience for Meningioca). Meningioca was designed by a multiprofessional core team comprised of family pediatricians (end-users), professionals with experience and skills in online or remote learning, and professionals with documented scientific expertise in vaccines and vaccinations, through a design-thinking approach [16].

For Meningioca, the design-thinking approach began with understanding the needs of the end-users (ie, PCPs). To ensure all required information was included, a map of topics discussed during health checks was generated (Textbox 1). Unique ideas to communicate this information to end-users in an innovative and engaging way were discussed, and gamification was identified as a potential solution. The map of health check topics was then adapted to suit a serious game, forming the basis of Meningioca’s content.

Textbox 1. **Contents discussed during each health check-up formed the basis of Meningioca’s modules. Each health check is intended to occur at a specific age. The contents of each health check are in line with this age and aim to monitor the physiological development of children and inform parents about preventable diseases.**

**Health check 1**

- Tasks to be done
- Vaccination status
- Meningitis B – characteristics and diagnosis
- Early autism diagnosis
- Communicate with the caregiver
- Nutrition and breastfeeding

**Health check 2**

- Tasks to be done
- General culture
- Vaccination status
- Hunger signals
- Meningitis B – lethality and disability
- Communicate with the caregiver

**Health check 3**

- Tasks to be done
- General culture
- Meningitis B – symptoms
- Vaccination status
- External body obstruction
- Communicate with the caregiver
- Weaning

**Health check 4**

- Tasks to be done
- Meningitis B – doses and serogroups
- Vaccination status
- Neurodevelopmental assessment of the child
- Communicate with the caregiver
- Babbling and language development

**Health check 5**

- Tasks to be done
- Child's oral hygiene
- Vaccination status
- Meningitis B – spread and transmission
- Risks of using digital devices
- Live attenuated virus vaccination
- Communicate with the caregiver

**Health check 6**

- Tasks to be done
- Scoliosis and posture
- Vaccination status
- Meningitis B – permanent disabilities
- Communicate with the caregiver
- Abilities at every age group

**Health check 7**

- Tasks to be done
- Sense of risk
- Vaccination status
- Meningitis B – contagion in adolescence
- General knowledge (adolescent language)
- Communicate with the caregiver
- Sexual experience

### Game Design

Meningioca was developed using Storyline 360 (a suite of Articulate 360), an authoring software, SCORM 1.2 (standard format), and TalentLMS, a learning management platform [17-19]. Participants play as an avatar of a goose, inspired by the Game of the Goose (a well-known board game that originated in Italy in the 15th century) and take on the role of a PCP. Using a narrative approach, players participate in a series of activities and mini-games, using and improving vaccination knowledge. Examples of the mini-games include a drag-and-drop activity, where users separate actions they would and would not carry out during health checks, as well as a quick-fire selection game, where users scroll through a video and click on correct answers to questions posed. Participants received points and feedback after each mini-game, with points gained the first time a module is played and players ranked against all other participants. Once each module had been unlocked, it was freely accessible for review or to refresh training. All data included in Meningioca are supported by a reference, either from scientific or gray literature, to ensure all information is confirmed with a reliable source.

### Game Refinement and Structure

**Figure 1.**
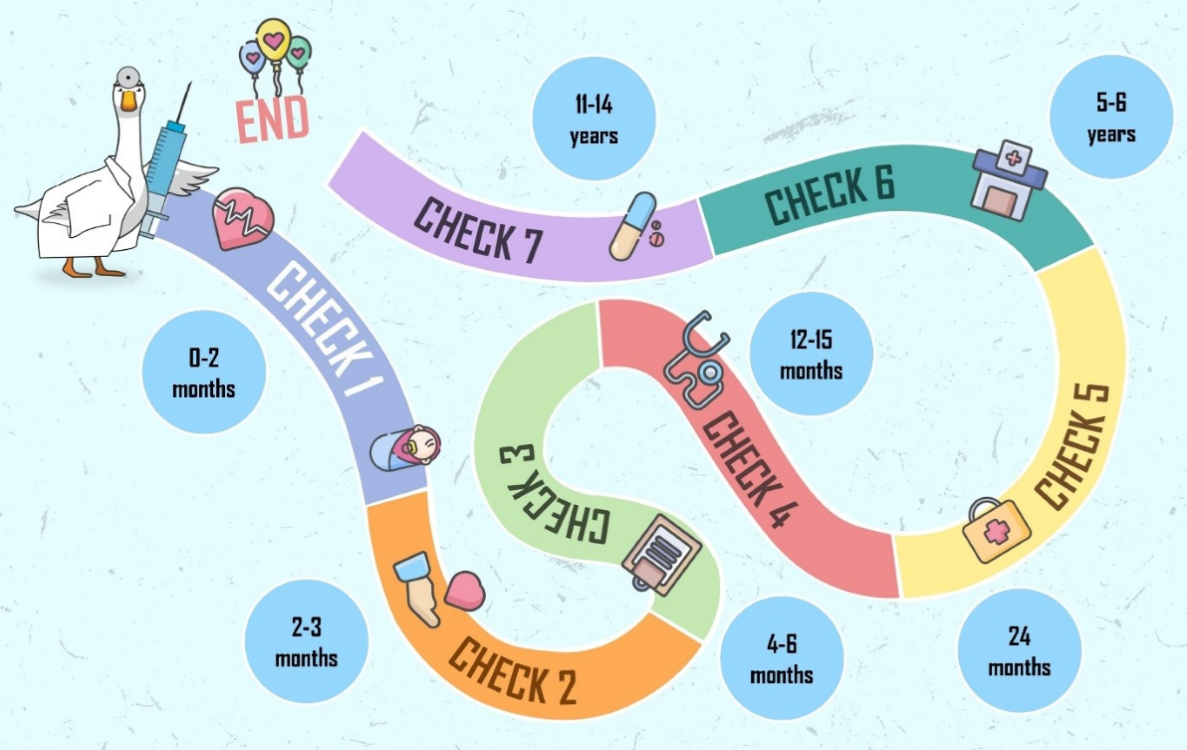
Meningioca modules.

A prototype of one module was played by 10 PCPs. Feedback from these testers suggested that the game was enjoyable, and further modules were produced. At launch (March 2023), Meningioca was comprised of 6 modules, with each module focusing on a different age group (0‐2, 2‐3, 4‐6, 12‐15, 24, and 60‐72 mo of age), selected to be in line with the Italian national immunization calendar. In April 2024, a 7th module focusing on adolescents (11‐14 y of age) was added (Figure 1).

**Figure 2.**
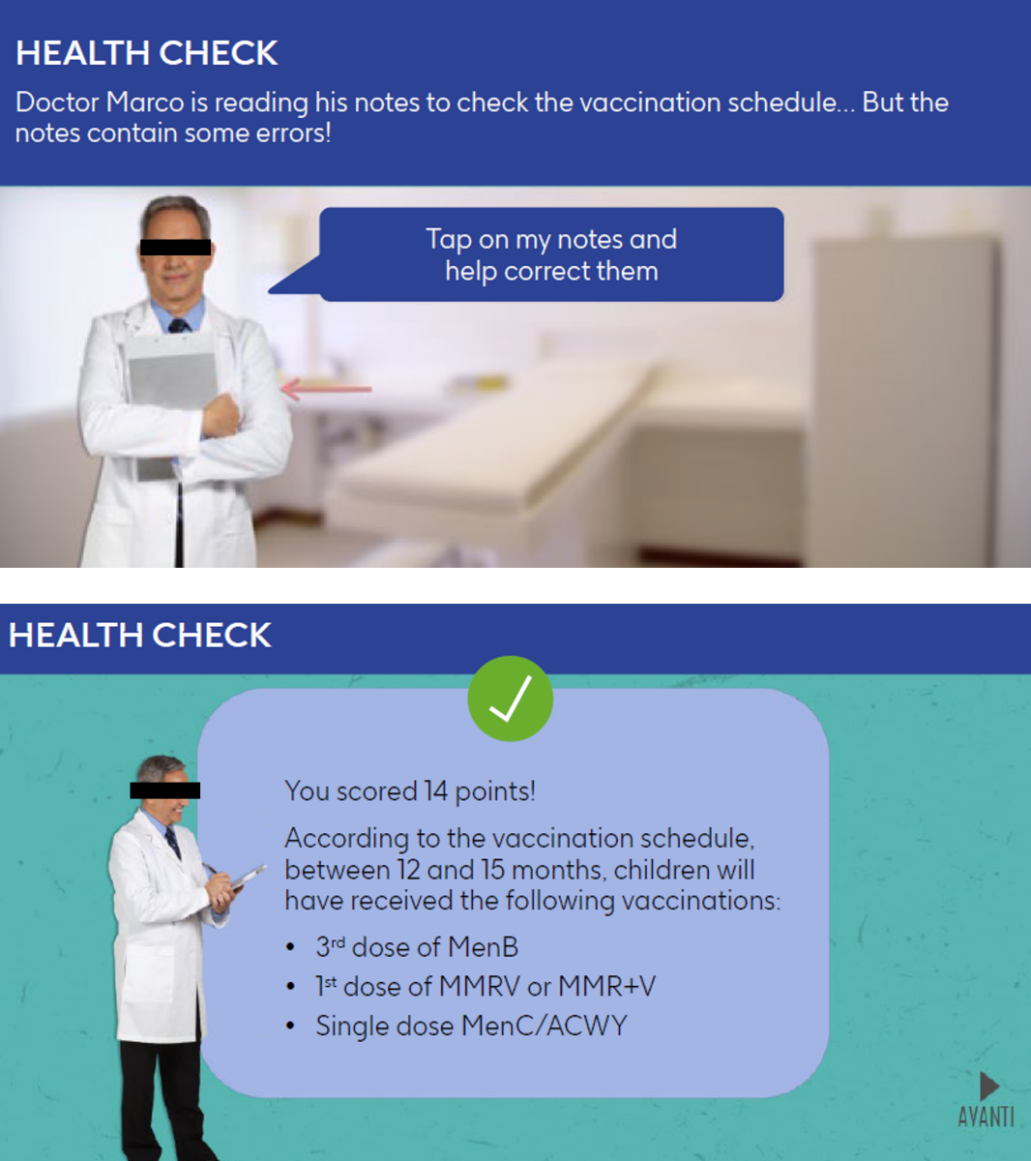
Example scenes from Meningioca depicting interactions with colleagues. Text from the game has been translated from Italian into English.

**Figure 3.**
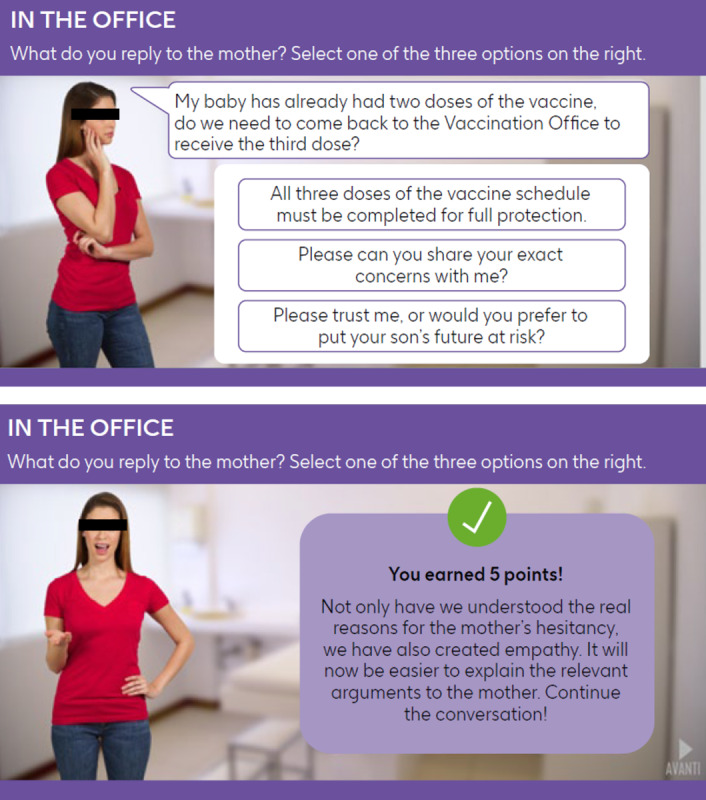
Example scenes from Meningioca depicting interactions with caregivers. Text from the game has been translated from Italian into English.

All modules within Meningioca are based on a fixed sequence of topics, mirroring discussions that could be expected during health checks for each age group. In brief, the following main themes are covered: health checks in general, child growth or development, vaccination calendar and immunization status check, deepening MenB understanding, communicating with the parent, and Italian general culture. The modules use elements of surprise via quizzes and involve role-playing scenarios where users interact with avatars of colleagues (Figure 2) and caregivers (Figure 3).

### Game Launch

At the launch of Meningioca in March 2023, members of the FIMP were invited to play the game via an email link. Members who subscribed to the game were notified of the launch of the module in April 2024. The effectiveness of Meningioca to educate PCPs on the value and proper timing of vaccination with a focus on MenB was measured via feedback questionnaires at the end of each module (Multimedia Appendix 1).

### Ethical Considerations

Meningioca was an educational project, not involving a human subject scheme nor collecting human data. Therefore, this project did not require ethics review as per the Linea Guida per la classificazione e conduzione degli studi osservazionali sui farmaci (GU Serie Generale n.76 del 31-03-2008) [20].

## Results

**Figure 4.**
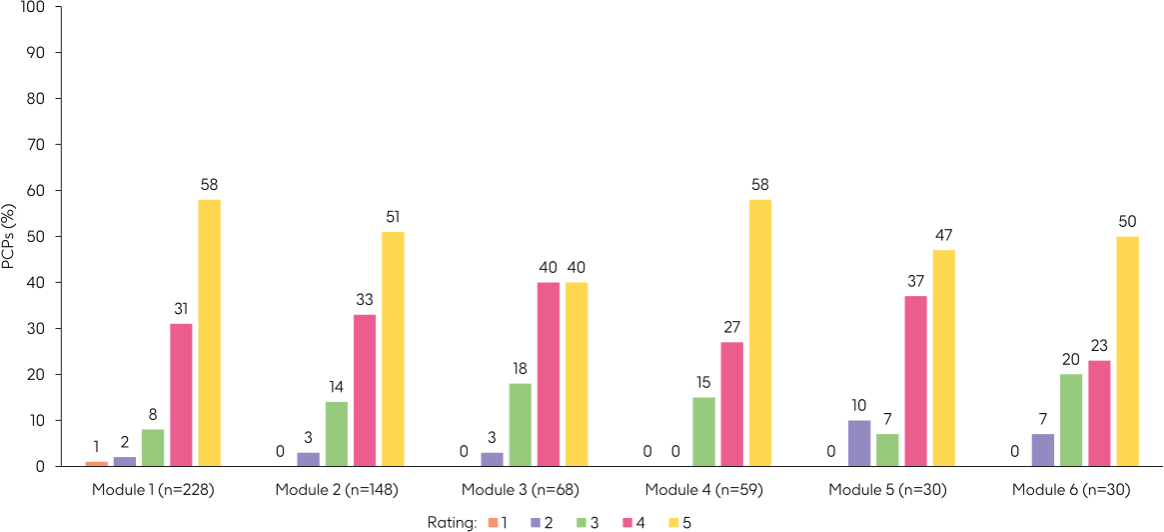
Proportion of PCPs rating each module on a scale of 1‐5 from March 2023 to September 2023. At the end of each module, PCPs were asked to rate how much they liked the module on a scale of 1‐5, with 5 as the highest score. PCP: primary care pediatrician.

Between March 2023 and May 2024, 471 PCPs accessed Meningioca and 1206 modules were completed, comprising 482 hours of learning. After Module 6, players were asked to rate their enjoyment of Meningioca on a scale from 1 to 5, with 5 being the highest score. Meningioca received a mean rating of 4.4/5. Participants were also asked to rate individual modules at the end of Modules 1‐6, and a breakdown of ratings is reported in Figure 4.

**Figure 5.**
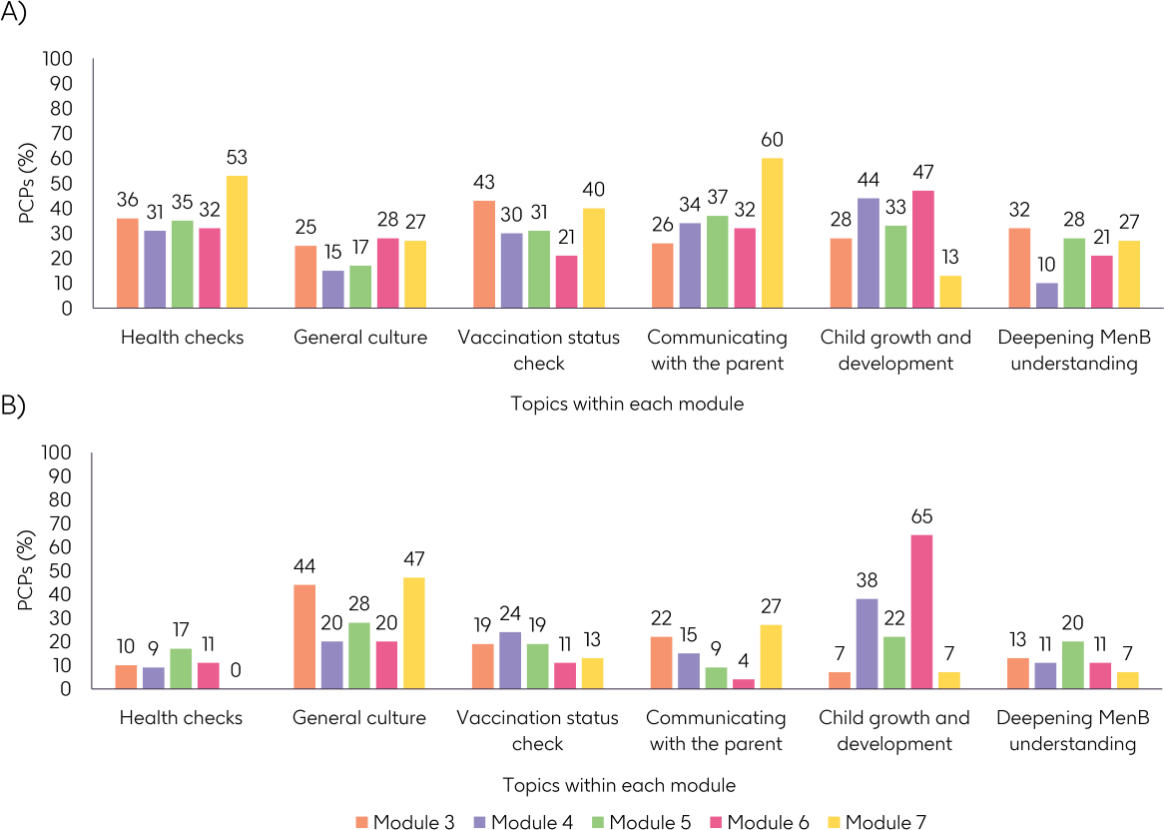
Proportion of PCPs that rated different topics within Meningioca as (**A**) the topic they liked the most and (**B**) the topic they found the most difficult. At the end of Modules 3‐7, PCPs were asked to choose their favorite topic within the module and the topic they found most difficult. MenB: serogroup B meningococcal disease; PCP: primary care pediatrician.

After Modules 3‐7, participants were asked to select their favorite topic and the topic that they found most difficult. There was a relatively even split regarding participants’ favorite topics; “communicating with the parent” and “health checks” received the most votes and “general culture” received the fewest (Figure 5A). “General culture” and “child growth and development” were voted as the most difficult module topics (Figure 5B).

On completion of certain modules, participants were asked whether they would recommend Meningioca to a colleague. Overall, 93% (n/N=63/68) answered “yes” at the end of Module 3, 90% (n/N=53/59) at Module 4, 90% (n/N=53/59) at Module 5, with the remaining participants answering “No.” Additionally, at the end of Module 3, participants were asked to what extent Meningioca could be considered a teaching tool to refresh/strengthen knowledge, with a response scale from “very little” to “very much”; 43% (n/N=29/67) of participants answered “a lot” and 32% (n/N=21/67) of participants answered “very much”.

## Discussion

### Principal Findings

To the best of our knowledge, Meningioca is the first gamified educational training tool developed within the field of vaccination, specifically designed for PCPs in Italy. The primary aim of Meningioca is to increase PCPs’ knowledge on the importance of health checks within the context of childhood vaccination and, on a larger scale, boost the below-target MenB vaccination coverage in Italy and decrease general vaccine hesitancy among parents. Early results from the first 14 months of use indicate that Meningioca is not only an enjoyable game but also serves as an effective teaching tool to refresh and strengthen vaccination knowledge.

### Comparison to Prior Work

Meningioca adds to the rapidly expanding field of digital vaccination-based serious gamification tools. Over the past 10 years, digital learning methods aimed at enhancing vaccination knowledge and programs have transitioned from being limited in use to becoming well established, with this evolution evidenced by the increasing number of these games available [14,15,21]. Meningioca, like many vaccine-based digital gamification tools, was aimed at a specific population [22,23]. Understanding the needs of the end-user is key for an engaging learning tool, and by focusing on one target group, the effectiveness of digital gamification tools can be maximized. PCPs were closely involved in the development of Meningioca, both as part of the initial multiprofessional design team and as testers of the pilot module. Involving the end-user from an early stage of development is a valuable strategy for tailoring the needs of a game and may have contributed to players’ enjoyment of Meningioca [15]. Furthermore, Meningioca reinforces the idea that digital gamification tools can be effective even in cohorts with a high level of base knowledge, as demonstrated in a previous study involving antibiotics and vaccination games, where senior students’ knowledge improved despite already possessing high baseline knowledge in these areas [22]. At present, the majority of vaccine-based digital gamification tools focus on one type of disease, such as influenza or human papillomavirus [15]. While Meningioca focuses on MenB vaccination, it also includes information on other childhood vaccinations, topics discussed during health checks, and general Italian culture. By including information on multiple childhood vaccinations, Meningioca ensures PCPs gain interconnected knowledge between different vaccinations, which may lead to a more comprehensive understanding and an increased ability to apply this knowledge more broadly. The web-based nature of Meningioca enhances its ease of use, as no downloadable content or additional devices are required to participate. While the impact of the type of platform used to deliver the tools, eg, web-based, mobile, or virtual reality has not currently been evaluated in terms of user preference, this web-based approach aligns with the majority of vaccine-related digital gamified tools [15].

Feedback from Meningioca’s first year indicated that the game is well-rated among players and enjoyable, as evidenced by Meningioca’s mean rating of 4.4/5. The even split between players’ favorite topics within modules suggests that the game offers a balanced experience, with each aspect developed to a high quality throughout. Enjoyment has been shown to have a positive effect on learning and memory, suggesting that Meningioca may be more effective at increasing knowledge if players enjoy it [24]. “Communicating with parents,” consistently one of the PCPs’ favorite topics, was voted as one of the least difficult, suggesting enjoyment and difficulty may be linked. As no single topic received an overwhelming majority of votes as the most difficult, it could be implied that players of a digital gamification tool need to experience a balanced level of challenge. This balance is crucial for effective learning, as players should feel challenged enough to engage in the game’s content, but not too much that it becomes overwhelming. Overall, early results suggest Meningioca was well received and is an effective tool to increase vaccination knowledge.

In Italy, regular health checks from birth to the age of 14 years are recommended to monitor the physiological development of children and inform parents about preventable diseases, including raising awareness of these diseases and encouraging caregivers to ensure their children receive available vaccinations. Nonetheless, many PCPs do not proactively schedule these appointments, potentially leading to gaps in preventive care and lapses in vaccination schedule adherence, ultimately impacting the child’s overall immunization profile [10]. Enhancing PCPs’ knowledge on the importance of vaccination may not only encourage them to proactively arrange future health checks but also improve their ability to effectively communicate this information to parents, thereby reducing parental vaccine hesitancy, fostering increased vaccine adherence, and helping to prevent the spread of disease. While it is not possible to measure this directly, by encouraging both PCPs to carry out more health checks and parents to take their children to receive vaccinations, Meningioca could boost childhood vaccination uptake, potentially leading to Italy meeting its national MenB vaccination coverage target.

### Strengths and Limitations

The ability of Meningioca to act as an effective teaching tool was strengthened by the inclusion of PCPs in its development. A large number of Italian PCPs were invited to play the game, improving the game’s generalizability and aiming to ensure that the players accurately represent PCPs overall. Despite these strengths, Meningioca was associated with limitations. First, feedback questionnaires were not consistent across modules, leading to data gaps when synthesizing responses. Consistent questions across modules may help in building a more comprehensive view of the perceptions of end-users. Second, the impact of Meningioca, in terms of its ability to reduce caregiver vaccine hesitancy and increase vaccine uptake, cannot be directly measured. Surrogate measures such as enjoyment and Meningioca’s use as a learning tool could instead be used as proxies.

### Future Directions

Further feedback from users will be essential for the improvement of Meningioca as a tool to increase vaccine knowledge among Italian PCPs. Meningioca will continue to be a live game for PCPs to access, in order to increase their knowledge on health check activities as well as their knowledge on vaccinations. Specific questions that test PCPs’ vaccine knowledge before and after using the training tool may help to quantify the effectiveness of Meningioca as a resource for increasing PCPs’ knowledge. Further validation will need to be carried out to assess whether Meningioca could be effective in reducing caregiver vaccine hesitancy and increasing MenB vaccine uptake.

### Conclusions

Digital gamification tools are an innovative and promising option for developing vaccination-related learning tools. Meningioca is an enjoyable and potentially effective educational resource for increasing PCP knowledge on the value and proper timing of vaccination, particularly MenB vaccination.

## Supplementary material

10.2196/70049 Multimedia Appendix 1 Questions that Meningioca users were asked following each module.

## Abbreviations

FIMP: Italian Federation of Pediatricians
IMD: invasive meningococcal disease
MenB: serogroup B meningococcal disease
PCP: primary care pediatrician
