# Supplementary material for: Increasing Vaccination Awareness for Italian Primary Care Pediatricians: Game Design and Usability Study
Source: JMIR Pediatr Parent. 2025 Nov 3;8:e70049. doi: 10.2196/70049 (PMC12582570; doi:10.2196/70049)
Supplement: Multimedia Appendix 1 [file pediatrics-v8-e70049-s001.docx]

Questions Meningioca users were asked following each module

| Question | Possible answers |
| --- | --- |
| Question following Modules 1–6 | |
| On a scale of 1 to 5, rate how much you liked Modules 1–6 | 1 |
|  | 2 |
|  | 3 |
|  | 4 |
|  | 5 |
| Questions following Module 3 | |
| Do you think Meningioca is a valid learning tool? | (Free-text answer) |
| How much can Meningioca be considered a teaching tool to refresh/strengthen knowledge? | Very little |
|  | A little |
|  | Somewhat |
|  | A lot |
|  | Very much |
| Do you find educational gaming a new and engaging way of learning? | Yes |
|  | No |
| Question following Modules 3–5 and 7 | |
| Would you recommend Meningioca to a colleague? | Yes |
|  | No |
| Questions following Modules 3–7 | |
| Which topic within Modules 3–7 did you like the most? (You can select more than 1 answer) | Health checks |
|  | General culture |
|  | Vaccination status checks |
|  | Communicating with the parent |
|  | Child growth and development |
|  | Deepening MenB knowledge |
| Which topic within Modules 3–7 topic did you find the most difficult? (You can select more than 1 answer) | Health checks |
|  | General culture |
|  | Vaccination status checks |
|  | Communicating with the parent |
|  | Child growth and development |
|  | Deepening MenB knowledge |
| Do you have any suggestions to share with us? | (Free-text answer) |
| Question following Module 6 | |
| On a scale from 1 to 5, how much did you enjoy Meningioca? | 1 |
|  | 2 |
|  | 3 |
|  | 4 |
|  | 5 |
